# Firm insoles effectively reduce hemolysis in runners during long distance running - a comparative study

**DOI:** 10.1186/1758-2555-3-12

**Published:** 2011-06-09

**Authors:** Kamal Janakiraman, Shweta Shenoy, Jaspal Singh Sandhu

**Affiliations:** 1 College of Physiotherapy, Cherraans institute of health sciences, 521 Siruvani main road, Telungupalayam Pirivu, Coimbatore - 641039, Tamilnadu, India; 2 Department of Sports Medicine and Physiotherapy, Guru Nanak Dev University, Amritsar, Punjab, India; 3 Department of Sports Medicine and Physiotherapy, Guru Nanak Dev University, Amritsar, Punjab, India

**Keywords:** Impact forces, intravascular hemolysis, insole hardness, footstrike

## Abstract

**Background:**

Shock absorbing insoles are effective in reducing the magnitude and rate of loading of peak impact forces generated at foot strike during running, whereas the foot impact force during running has been considered to be an important cause of intravascular hemolysis in long distance runners. Objective of this study was to evaluate the intravascular hemolysis during running and compare the effect of two different types of insoles (Soft and Firm) on hemolysis.

**Methods:**

Twenty male long and middle distance runners volunteered to participate in this study. We selected two insoles (Soft and Firm) according to their hardness level (SHORE 'A' scale). Participants were randomly assigned to the soft insole (group 1) and firm insole (group 2) group with ten athletes in each group. Each athlete completed one hour of running at the calculated target heart rate (60-70%). Venous blood samples were collected before and immediately after running. We measured unconjucated bilirubin (mg/dl), lactate dehydrogenase (μ/ml), hemoglobin (g/l) and serum ferritin (ng/ml) as indicators of hemolysis.

**Results:**

Our study revealed a significant increase in the mean values of unconjucated bilirubin (P < 0.05) while running with soft insoles indicating the occurrence of hemolysis in this group of athletes. Graphical analysis revealed an inverse relationship between hardness of insoles and hemolysis for the observed values.

**Conclusion:**

Our results indicate that intravascular hemolysis occurs in athletes during long distance running and we conclude that addition of firm insoles effectively reduces the amount of hemolysis in runners compared to soft insoles.

## Background

Over the course of a one mile run an individual makes approximately 1600 foot strikes [1,2]. At the time of contact between foot and ground, impact forces and pronation forces places a large stress on the structures of lower extremity [2-4] and the exposure to repeated impact loading was linked to the development of runners' injuries [5,6].

Shoe insoles play an important role in the reduction of ground reaction force, especially during running [7]. Running related injuries are, to some degree, caused by excessive peaks in the impact phase of the ground reaction force [8] and it has been suggested that shock absorbing insoles reduce the magnitude and rate of loading of the peak forces generated at foot strike during running and walking, and reduce the ground reaction forces across the foot [9].

Long distance running is associated with a wide range of significant changes in vascular volumes [10-12]. Research has identified intravascular hemolysis as an important cause of alteration of vascular volumes in long distance runners, whereas force impact during foot strike has been suggested to be an important mechanism of intravascular hemolysis [14,15] during running. Few studies have focused on the effect of insole materials on vascular parameters during running [16-19] and the results among them were contradictory.

Importance of this iron depletion state is that any sufficient decrease in hemoglobin (Hb) has a proficient effect on total VO_2 _max which in turn can hinder the performance of the athletes [20]. The iron depletion state may be significant in elite distance runners even in the absence of anemia (Martin et al 1980), which shows the importance of hemolysis [21]. This decreased oxygen carrying capacity in the blood can also lead to increase in the lactic acid build up and prolonged recovery. The importance of intravascular hemolysis is that it may lead to sports anemia [22].

Single episode of hemolysis may not cause anemia, but daily or twice daily episodes of hemolysis can result in an iron depletion status. This iron depletion status can evolve in to an iron deficiency anemia, which in turn can affect the performance of the athletes.

Increase in the levels of unconjucated bilirubin is an important indicator of intravascular hemolysis. The elevation of unconjucated bilirubin is due to the fact that bilirubin is one of the principal heme catabolite and hence its level is an index of the rate of red blood cell (RBC) destruction. Since hemolysis results in excessive heme catabolism it results in increased serum bilirubin. The elevation of Lactate dehydrogenase (LDH) is another indicator of intravascular hemolysis. LDH has long been considered a useful clinical marker of intravascular hemolysis. Its serum levels are mildly elevated in extravascular hemolysis, such as immune hemolytic anemia, but are substantially elevated with intravascular hemolysis [23]. Iron deficiency after prolonged running have been reported and indicates hemoglobin and serum ferritin as an important indicators of iron status in athletes [24-26].

Consequently, the purpose of our study was to evaluate the effect of two insoles of different hardness (soft and firm) on hemolysis. We chose the hardness property of insoles based on previous work validating the effect of this variable in reducing impact force during running [9,27,28]. We speculated that addition of firm insoles would effectively reduce the foot impact forces during running [5,6,28,29]. Specifically, we hypothesized that addition of firm insoles would result in lesser hemolysis compared to soft insoles.

## Methods

### Participants

The participants for this study were 20 male long and middle distance runners. The sample size was calculated by using sample size software (nMaster 1.0, Christian Medical College, Vellore, India)). All the participants were screened by a Physical activeness readiness questionnaire (PAR-Q). Female runners and athletes under regular iron supplements were excluded from the study. All the participants were instructed in the data collection procedure and then signed an informed consent form that was approved by the institutional ethical committee, which also approved the study procedure.

### Grouping of participants

The participants were randomly assigned in to two groups (using online research randomizer software -Version 4.0) with ten athletes in each group (*Power *= 0.24, *Effect size = *0.67). Grouping of athletes were made as follows: Group 1 - Participants running in their regular shoes with soft insole inserts (*n *= 10) and Group 2 - Participants running in their regular shoes with firm insole inserts (*n *= 10).

The anthropometric characteristics (Means ± SD) of the athletes were: Group 1: age (20.4 ± 1.43 years), height (1.65 ± 0.08 m), weight (58.5 ± 5.25 kg) and body mass index (21.46 ± 0.75), Group 2: age (21 ± 2.08 years), height (1.7 ± 0.02 m), weight (61.3 ± 3.5 kg) and body mass index (21.22 ± 0.9).

### Procedures

#### Hardness testing of shoe insoles

Nine pair of shoe insoles were randomly purchased and tested for their hardness using SHORE 'A' SCALE in a material testing lab of a central government factory (as a measure of confidentiality, as requested by the concerned officials, the name and location of the factory is not mentioned). Later four insoles were selected from the nine insoles according to hardness level and retested at 'Footwear Testing Laboratory' Shoe Design and Development Centre, CENTRAL LEATHER RESEARCH INSTITUTE (CLRI), Adyar, Chennai. The test had been conducted at 20°C and 65% RH (relative humidity) and the test method used was SATRA TM 206:1999. From the results of hardness testing finally two insoles were selected and classified in to firm (sole A) and soft (sole D) insole according to their hardness (Table 1).

**Table 1:** Hardness of Insoles - SHORE 'A' Scale

Insoles	Hardness
Sole - A*	44 - 49
Sole - D†	32 - 36

* Sole - A categorized as firm insole

† Sole - D categorized as soft soles

#### Running protocol

Each athlete reported to the exercise physiology laboratory before the actual running protocol. The physical characteristics (age, height, weight) of all the athletes were measured and target heart rate (THR) (60-70%) was calculated, which was set as the intensity for the running protocol. A further condition was that no intense training was carried out 24 hrs before running trial.

The standardized running protocol began a day after the initial evaluation. All athletes wore medium-weight training shoes of a similar type, with all shoes in good condition. Running protocol consists of 5 minutes of jogging as warm up at 40-50% of THR. After the warm up period each athlete completed one hour of running either with firm insoles (Group 1) or soft insoles (Group 2) at their calculated THR (60-70%).

Heart rate monitoring: Heart rate was measured at regular intervals (every 15 mins) after which adjustments were made if necessary to the speed. Heart rate was monitored by athlete and the investigator using a polar heart rate monitor (PC coach light, version 3.0.3, Biometrics inc. Boulder. Co, USA).

### Blood sample collection

Blood samples were collected before and immediately after running. At each sample point, the blood was collected via veni puncture of the ante cubital vein, with the athlete in a sitting position. To control for plasma volume shifts due to postural change, sampling before and after running was carried out in an identical fashion, with the athlete in a sitting position. Extreme care was taken during the collection of blood samples to avoid the possibility of hemolysis. Two milliliters of blood were collected in an Ethylenediaminetetraacetic acid (EDTA) tube for hemoglobin (Hb) estimation. Two milliliters of blood were transferred into a serum tube and allowed to clot at room temperature. The blood samples were stored in the ice box and was immediately transferred to the laboratory (situated 200 m from the ground) and refrigerated. The serum clotted samples were centrifuged at 3000 revolutions per minute (RPM) for 10 minutes. The blood samples were evaluated within 24 hours after the collection.

The blood parameters which were used to measure the hemolysis are unconjucated bilirubin, LDH, Hb and serum ferritin. Blood samples were analyzed using minitechno-biochemistry analyzer (Lamicoat international, Italy, 2007) for unconjucated bilirubin, LDH and Hb estimation and ELISA-APR microplate reader (Logotech-ISE group, Italy, 2007) for serum ferritin estimation. Blood analyses were carried out by an experienced laboratory technician and according to the procedure guidelines given for the individual kit.

### Statistical analysis

The statistical analysis involved non parametric data evaluation as the observed value did not satisfy the criteria for parametric test evaluation. Wilcoxon signed rank test was used to evaluate the level of hemolysis between pre and post run for both groups and a Mann - Whitney U test to evaluate the significance between firm and soft insoles on hemolysis. The significance level was set at *P *≤ 0.05. All the values are represented as means ± SD. The power of the study was calculated using power and sample size calculator (PS version 3.0.5). SPSS statistical software (version 17.0) was used to compute all the data analyses.

## Results

Significant hemolysis was observed in participants who ran with soft insoles (*P *= 0.017). Unconjucated bilirubin levels increased significantly post run in these runners (Z (18) = 2.397, *P *= 0.017) indicating the occurrence of hemolysis. However there were no significant differences observed (between pre and post values) in the mean values of unconjucated bilirubin (Z (18) = 1.26, *P *= 0.208) while running with firm insoles. Similarly there were no significant differences in the mean values of other hematological variables while running with soft insoles [LDH (Z (18) = 0.051, *P *= 0.959), Hb (Z (18) = 0.154, *P *= 0.878) and serum ferritin (Z(18) = 0.459, *P *= 0.646) ] and with firm insoles [LDH (Z (18) = 0.097, *P *= 0.333), Hb (Z (18) = 0.102, *P *= 0.919) and serum ferritin (Z (18) = 1.070, *P *= 0.285) ].

For each of the hematological variables, the interaction between soft insoles and firm insoles was not significant [Unconjucated bilirubin (Z (18) = 1.400, *P *= 0.161), LDH (Z (18) = 0.681, *P *= 0.496) Hb (Z (18) = 0.076, *P *= 0.939) and serum ferritin (Z (18) = 0.529, *P *= 0.597)].

**Figure 1:**
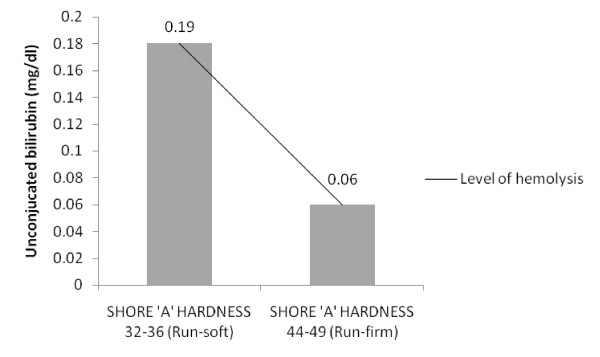
**Comparison between hardness of insoles and unconjucated bilirubin**.

**Figure 2:**
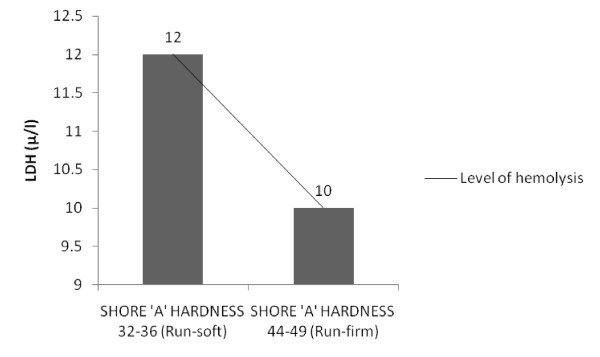
**Comparison between hardness of insoles and LDH**.

Graphical analysis revealed an inverse relationship between hardness of insoles (SHORE 'A' scale) and hemolysis (Figure 1 &2) for the given data.

## Discussion

This study was conducted for the purpose of examining hardness property of two different shoe insoles (Soft and Firm) on intravascular hemolysis during running. Expected changes in the values of unconjucated bilirubin (*P*<0.05) were observed in athletes who ran with soft insoles, indicating the occurrence of hemolysis in this group of athletes. The findings concord with the results of previous studies which state that intravascular hemolysis occur in long distance runners [17,18] and suggest that there is a direct relationship between serum bilirubin and intravascular hemolysis in long distance runners [23,30].

There were no significant differences in the degree of hemolysis incurred between two insole inserts (*P*>0.05). However, participants who ran with soft insole insert demonstrated significant increase in the mean values of unconjucated bilirubin (mg/dl) (*P*<0.05), when compared to athletes who ran on firm insole insert (*P*>0.05) signifying the occurrence of greater hemolysis in this group of athletes. Although LDH values did not demonstrate any significant variations in both group athletes (Table 2), there was an increase in the mean values of LDH (μ/l) post run in athletes running with firm insoles (mean = 12 ± 55) compared to athletes running with soft insoles (mean = 10 ± 93). Previous researchers have emphasized the role of shoe insoles in the shock absorption and reduction of impact forces during running [7], which provide a rationale for the changes observed in our present study.

**Table 2:** The Variations of Hematological Parameters in Both Group Participants (Mean ± SD)

	Group 1(Soft sole) (*n *= 10)		Group 2 (Firm sole) (*n *= 10)	
	**Pre run**	**Post run**	**Pre run**	**Post run**

UBR (mg/dl)	0.68 ± 0.67	0.87 ± 0.84*	0.37 ± 0.15	0.43 ± 0.19
LDH (μ/ml)	319 ± 201	331 ± 193	381 ± 190	391 ± 183
Hb (g/l)	13.3 ± 1.6	13.1 ± 1.8	13 ± 1.9	13.01 ± 1.71
FER (ng/ml)	67.3 ± 62.1	66.9 ± 64.8	72.4 ± 41.02	71.1 ± 40.5

* *P *< 0.05 (significance measured by Wilcoxon signed rank test)

Conversely, the results of this present study appeared to contradict reports of other authors [16-19]. There had been contradicting reports regarding the role of insoles on hemolysis. Initially it was suggested that hemolysis occurred more while running with firm insoles compared to soft insoles [16,17] On the contrary, recent research findings observed insignificant hemolytic changes while running with different insoles [18,19] However, all the authors shared a common view point that intravascular hemolysis does occur in the runners and addition of insoles was effective in blunting the hemolytic episodes.

Although the present study demonstrated contradictory results to previous studies suggesting greater hemolytic changes while running with soft insoles when compared to firm insoles, an in-depth review of impact force behaviors and shock absorption characteristics of insoles during running reveals some important facts, which support the findings of our present study. Vertical force impact peak and vertical force propulsive peak were reported to be high in soft soles compared to harder sole [31] and it was suggested that forefoot impact force is influenced by sole thickness and hardness [28] whereas, a study reported that peak ground reaction force was significantly greater in normal and hard midsoles compared to soft midsoles [32]. Based on these considerations and on earlier results [29,33] the relationship between hardness of two insoles on hemolysis was analyzed. Graphical analysis revealed an inverse relationship between the hardness of insoles (SHORE 'A' scale) and hemolysis (unconjucated bilirubin mg/dl (Figure 1), LDH μ/l (Figure 2)), suggesting that greater hemolysis occurred with lesser hardness and the hemolytic status reversed as hardness of insoles increased. Tomita et al [27] related shock absorption and SHORE hardness in their study and confirmed that there is a correlation between hardness and maximum impact force in the materials that showed shock absorption by elastic deformation. Consequently, it was assumed that less hardness of the shoe insoles resulted in larger impact force acting on the foot, resulting greater hemolysis in these runners. But this relationship may not be true if the other extreme is considered i.e., hard insoles. Previous research [29] suggested that both too soft and too hard of a sole can be detrimental to shock absorption and that a good, medium hardness is recommended for optimal shock absorption, a fact that support the results of this present study. These observations indicate that shoe insoles do play an important role in reducing the amount of intravascular hemolysis in athletes during running. The results of this present study concord with the previous observations which states that the material properties of running shoes correlate with physiological measurements and appropriate cushioning reduces the RBC abnormalities experienced in long distance runners [16,17].

Limitations of this study exist primarily with regard to the insole properties examined. We believe that inclusion of measurements of ground reaction force and shock absorption properties, and its relation to hardness of insole and hemolysis would have provided a better insight in to this current problem. Furthermore, this present study did not incorporate hard insoles which could have given us a better clarity to this phenomenon. We also felt that repeated measure design would have been better than two group design. Due to athlete's constraint and coach cooperation was not there to collect repeated blood sample, it was difficult to convince these local coaches in implementing repeated measure design. Future research examining the impact forces and shock absorption properties of insoles and their relation to hemolysis appears warranted.

### Clinical relevance

Intravascular hemolysis was thought to be a physiological adaptation to exercise [34,35], but prolonged strenuous training may disturb this conservative mechanism and repeated episodes of hemolysis over time may significantly impact runners. Although intravascular hemolysis may not lead to sports anemia or true iron deficiency anemia, it can result in an iron deficiency status [24], which may hinder these runners in achieving maximal aerobic power [25] and dull their performance [26,36]. Athletes and coaches must be aware of this potential problem and insist on wearing firm cushioned shoes to reduce the severity of hemolysis.

## Conclusions

Our observations support the supposition that intravascular hemolysis occurs in runners during long distance running. Furthermore, we found that hardness of the shoe insoles was inversely related to intravascular hemolysis for the values observed in our current study. Hence, we conclude that addition of firm insoles is an effective way of reducing the amount of intravascular hemolysis in runners compared to soft insoles during long distance running.

## Competing interests

We authors jointly declare that that we do not have any financial or non-financial competing interests in relation to our manuscript titled "Firm Insoles Effectively Reduce Hemolysis in Runners during Long Distance Running - A Comparative Study", submitted to the Journal of Sports Medicine, Arthroscopy, Rehabilitation, Therapy and Technology.

## Authors' contributions

KJ participated in design of the study, carried out running trials, involved in data collection and drafted manuscript. SS participated in implementation of research, performed statistical analysis and evaluated the drafted manuscript JSS participated in research design and coordination, conceived of the study and assisted in final drafting of manuscript All the authors read and approved the final manuscript
